# Does social support matter? The mediating links with coping strategy and anxiety among Chinese college students in a cross-sectional study of COVID-19 pandemic

**DOI:** 10.1186/s12889-021-11332-4

**Published:** 2021-07-02

**Authors:** Yue Li, Jun Peng

**Affiliations:** 1 Division of Art, Shenzhen University, Shenzhen, Guangdong Province China; 2 School of Education, City University of Macau, Macau, China

**Keywords:** Coping strategy, Social support, Anxiety, Mediator, COVID-19 pandemic

## Abstract

**Background:**

The provision of public adaptive coping strategies to reduce psychological tension during the ongoing COVID-19 pandemic is critical. We sought to provide evidence-based guidance for psychological intervention, exploring the potential mediating roles of three sources of social support (i.e., subjective support, family support and counselor support) between coping strategies (i.e., cognitive coping, emotional coping and behavioral coping), and anxiety among college students at the height of the pandemic in China.

**Methods:**

Using the Coping Strategy Questionnaire, Social Support Questionnaire, and Self-Rating Anxiety Scale, this large-scale online study analyzed the levels of social support, coping, and anxiety among 2640 college students in China from February 21st to 24th, 2020, when the students had been isolated at home for 1 month since the lockdown of Wuhan city.

**Results:**

Students reported high levels of cognitive coping, behavioral coping, and social support. They also experienced low levels of anxiety and emotional coping. Anxiety was significantly and negatively related to coping and social support. The mediating roles of three sources of social support were found between cognitive coping, behavioral coping, and anxiety, respectively. However, the effect of emotional coping on anxiety was not found to be mediated by social support.

**Conclusions:**

Adopting positive coping strategies may enhance social support that in turn relieves anxiety. The effect of social support, especially family and counselor support, should arouse greater awareness in coping with the pandemic cognitively and behaviorally.

**Supplementary Information:**

The online version contains supplementary material available at 10.1186/s12889-021-11332-4.

## Background

The outbreak of Coronavirus Disease 2019 (COVID-19) has invoked panic and anxiety in the world. Under this unprecedented circumstance, the Chinese government has taken an active stance and implemented extreme measures to mitigate the dissemination of the disease. Some of these measures include suspending all public emigration in Wuhan on January 23rd, 2020, introduction of forced quarantine for citizens in the peak period, extending holidays, and closing schools [1]. As a result, people resorted to adaptive coping strategies as well. A great deal of attention has been paid by citizens to COVID-19 epidemic news and overreaction triggered by rumors and exaggerated information occurs [2, 3]. In a survey conducted in Canada in early February 2020, 33% of respondents revealed little confidence in the capability of their healthcare system to handle the disease, indicating low emotional coping among individuals [4].

In addition to coping strategies, a range of negative psychological consequences is usually accompanied by physical harm during the outbreak of a pandemic, such as depression, panic, and anxiety [5]. A recent study stated that more than 70% of clinicians who treated confirmed COVID-19 patients had gone through psychological distress, and 44.6% had been experiencing anxiety under the daunting environment [6]. People who are required to quarantine are prone to various mental problems. Self-isolated adults who were suspected COVID-19 cases reported high levels of anxiety with low sleep quality [7]. The reason for this psychological pressure is primarily due to the lack of interpersonal association, and therefore, the significance of social support deserves consideration [8].

Stewart’s coping theory stated that social support should be determined as one of the coping resources [9]. Since seeking social support has been regarded as one of the most adaptive approaches to cope with stress during crises [10], a number of previous COVID-related studies have emphasized the need for social support. A study aimed at clinicians in Wuhan, China, who treated infected patients found that the social support of healthcare professionals had an indirect effect on sleep quality. However, most studies measured general social support rather than specific social support from various sources. To the best of our knowledge, a study sampled by 628 college students is the only one identifying the mediating role of multiple social support from family, friends, and romantic partners, respectively between stress and well-being [11]. Given the limited scope of available studies coupled with specific social support in this crisis, the present study focused on the roles of three sources of social support (i.e., subjective support, family support, and counselor support) in managing the COVID-19 pandemic.

Although a variety of studies have drawn attention to coping strategies, anxiety, and social support [12, 13], few studies have been conducted to identify their relationship under the circumstance of the COVID-19 pandemic. Moreover, to our knowledge, a large-scale empirical study aimed at college students’ mental health condition and coping in the pandemic seems insufficient. Furthermore, there is a lack of research on the role of various sources of social support in coping and anxiety. Therefore, the study aimed to examine the relationships between coping strategies, social support, and anxiety. Also, the study delved into the potential mediating effect of social support on these relationships to disentangle the internal support mechanisms underlying coping and mental health among Chinese college students in January and February when the epidemic peaked in China. The data at that time could reflect the authenticity and reliability of the students’ coping status, which highlighted the significance of this research. Moreover, our study is novel in probing into the mediating roles of differentiated sources of social support in coping and anxiety during this time.

## Methods

### Design and research questions

Using stratified random sampling in terms of students’ sex, major, and academic degree in this large-scale study, 2640 college students from a comprehensive university in Shenzhen, an important economic zone in China, completed the online survey during February 21st to 24th 2020, when the students had been isolated at home for at least 1 month since the lockdown of Wuhan city. The following research questions and hypotheses in the context of the COVID-19 pandemic are proposed.

- RQ1: What is the status of coping strategies, social support, and anxiety among Chinese college students?
- RQ2: What are the relationships between coping strategies, social support, and anxiety?

Given the mediating role of social support between performance and mental health, is it plausible that various sources of social support mediate the relationship between coping and anxiety during the pandemic? Based on the findings above, three hypotheses are proposed.

- H1: The relationship between cognitive coping and anxiety is mediated by social support.
- H2: Social support has a mediating effect on the relationship between emotional coping and anxiety.
- H3: Social support plays a mediating role in the relationship between behavioral coping and anxiety.

### Conceptual model

**Fig. 1:**
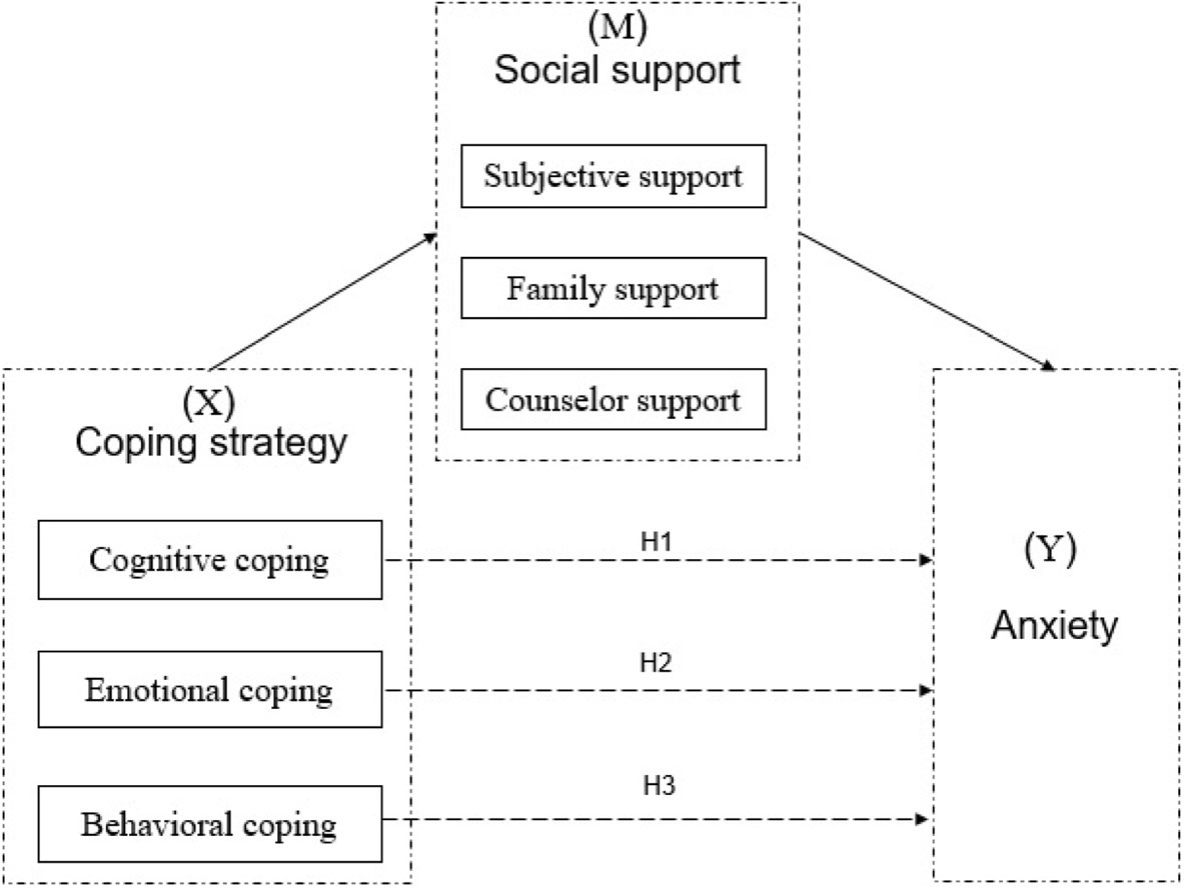
Note: Dashed lines indicate the mediating effect of coping strategy on anxiety

The conceptual model is presented as follows (Fig. 1):

Coping strategies are widely classified as either positive or negative [14]. However, general coping strategies are differentiated from practical coping strategies which refer to specific behaviors in the pandemic context [15].

To address pandemic coping more specifically, the present study adopted Lazarus and Folkman’s theory of psychological stress which divided coping into problem-focused coping (i.e., cognitive and behavioral efforts to solve the problem causing the distress) and emotion-focused coping confronted with stress [16], and classified coping into cognitive, emotional, and behavioral coping. Cognitive coping indicated the awareness of the current crisis and the way pandemic news was handled. Current COVID-19-related studies proved that proper perception of accurate information could predict lower levels of anxiety [5], and adequate information about the acquisition of the virus was negatively related to the emergence of mental problems [17]. Based on the themes drawn by the Cognitive-Motivational-Relational theory of emotion, emotional coping pertained to the emotional experience and adaptive actions that came from them [18], which in the present study referred to the affective uncertainty and worries about the disease. Evidence proved that more intolerance of uncertainty predicted greater reports of anxiety in the 2009 H1N1 pandemic [19]. In addition, Emotion-focused coping employed in the COVID-19 pandemic such as “being angry, and yelling” tended to highlight the sense of powerlessness and anxiety [17]. Behavioral coping signified the activities to alter the stressful encounter [16], indicating the pandemic precautions. Personal preventive measures such as hand washing and wearing face masks were found to be associated with fewer psychiatric symptoms [5, 20].

In the social support norm, subjective support denoted individual resilience targeting the stressors in the pandemic, which comprises personal competence and confidence in one’s instincts to cope with adverse circumstances [21]. Family support herein represented the perceived support quality from family. The Centers for Disease Control and Prevention argued the crucial role of family support in coping with anxiety [22]. Family care could be preferred as a critical source and it is imperative to facilitate meaningful communication between family caregivers and old adults in long-term care facilities [23]. Additionally, counselor support referred to emergent countermeasures implemented by psychological counselors, embracing the issue of self-help manuals, operation of 24-h psychological hotlines, and online survey applications [24]. Since the closing of schools, many universities in China have initiated online psychological counseling services and published mental health guidelines to support college students [25].

### Measures

#### Sociodemographic characteristics questionnaire (SCQ)

The SCQ was designed to collect students’ sociodemographic data, including sex, major, academic attainment (graduate or undergraduate), current location (Hubei Province, the pandemic zone or not), isolation condition (medically observed or not), and self-perceived health status.

#### Coping strategy questionnaire (CSQ)

The CSQ was used to evaluate coping strategies adopted by the students in the COVID-19 pandemic. This novel questionnaire was framed on the theory of psychological stress [16], and COVID-related coping was then divided into cognitive coping (CC; items 1–3), emotional coping (EC; items 4–6) and behavioral coping (BC; items 7–10) (see Table S1). The 5-point Likert scale was rated from “strongly disagree” to “strongly agree” with the subscale of emotional coping reversely coded, with higher scores indicating higher frequencies of positive coping.

Principal components analyses were conducted to verify the validity of this scale. The Kaiser-Meyer-Olkin test yielded an index of 0.795, and Bartlett’s test of sphericity was significant. Three factors emerged, accounting for a total of 61.396% of the variance, with the factor of CC, EC, and BC accounting for 24.540, 18.674, and 18.183%, respectively. The Cronbach’s alpha coefficient was 0.83 with 0.70, 0.67, and 0.77 for each subscale dimension (CC, EC, BC) respectively.

#### Social support questionnaire (SSQ)

The SSQ revised from the Chinese version of the SSQ was used to assess the levels of social support perceived by the students [26]. The questionnaire contained three dimensions, including subjective support (items 1–2), family support (items 3–4) and counselor support (items 5–6). Each item is rated from 1 to 5 (1 = strongly disagree, 5 = strongly agree), with a high score reflecting a high level of social support. In the present sample, with 0.89 for the overall SSQ, the Cronbach’s alpha coefficients for the subjective support, family support, and counselor support subscales were 0.93, 0.77, and 0.97, respectively.

#### Self-rating anxiety scale (SAS)

The anxiety of the students was measured using the SAS [27]. The SAS questionnaire contained 20 four-point-scale items, of which the aggregate score was then multiplied by 1.25, with higher scores indicating more severe levels of anxiety. The scale was reliable, with a Cronbach’s alpha coefficient of 0.89 in this research.

### Data collection procedures

This study was granted ethics approval by the Human Research Ethics Committee of City University of Macau (EA2001025). Eligible participants were young adults aged 18 to 25 years who are currently enrolling at a Chinese university. Of the 2680 participants recruited, 2640 students completed the survey, for a return rate of 98.5%. Subsequently, the data of 2640 participants were validly analyzed in this study. The participants were sent an email with the purpose and requirements for the investigation. After granting permission for inclusion, the respondents completed the questionnaires during February 21st and 24th of 2020 anonymously via an online survey with a random gift card distribution as incentives at the end of the questionnaires.

### Data analysis

The data were analyzed using IBM SPSS statistics 24.0 for Windows (IBM Corp., Armonk, NY, USA). First, descriptive statistics were used to summarize the demographic characteristics of the participants and the means of the investigated variables. To test the normality of data distribution, one-sample Kolmogorov-Smirnov test and histogram plot were used. Then, correlations between coping, social support, and anxiety were measured by Spearman’s correlation analyses. Finally, to explore the roles of social support in mediating the relationships between coping and anxiety, bootstrapping was applied with 5000 resamples with bias-corrected 95% confidence intervals (CI). In the mediation effect test, if the 95% CI did not contain zero, the mediation effect would be significant at the 0.05 level [28]. Meanwhile, both the direct and total effects were analyzed.

## Results

### Descriptive statistics

Demographic characteristics are presented in Table 1. The students surveyed were composed of graduates and undergraduates, some of whom were medically observed or isolated in Hubei Province, a province with a comparatively severe pandemic. In addition, the students’ ratings on perceived health condition were collected to assess their current self-efficacy on health.

**Table 1:** Sociodemographic characteristics of the participants (*n* = 2640)

Sociodemographic variables	Subcategories	n	%
Sex	Male	824	31.21
	Female	1816	68.79
Academic attainment	Graduate student	383	14.51
	Undergraduate	2257	85.49
Current location	Hubei Province (the epicenter)	62	2.35
	Other provinces	2578	97.65
Isolation condition	Medically observed	24	0.91
	Not isolated	2616	99.09
Self-perceived health status	Very healthy	2136	80.91
	Healthy	465	17.61
	Not sure	37	1.4
	Not healthy	2	0.08

### Correlation analyses

The means and standard deviations of coping, social support, and anxiety are given in Table 2. The students showed high levels of cognitive coping (M = 4.53, SD = 0.47), behavioral coping (M = 4.76, SD = 0.37), and social support (M = 4.59, SD = 0.55), and low emotional coping (M = 2.76, SD = 0.87). Compared with the Chinese norm of the SAS (M = 29.78, SD = 0.46) in the ordinary period, the students experienced significantly higher anxiety during the epidemic period (M = 36.25, SD = 4.57, t = 72.815, *p* = 0.000) [29].

**Table 2:** Correlations between coping strategy, social support, anxiety, and subscales

Variables	1	2	3	4	5	6	7	8	9
1. CS^a^									
2. CC	.560**								
3. EC	.694**	−.021	–						
4. BC	.505**	.462**	−.097**	–					
5. SS^a^	.421**	.531**	−.022	.572**	–				
6. SS^b^	.436*	.469**	.008	.553**	.782**	–			
7. FS	.399**	.507**	−.046*	.557**	.883**	.686**	–		
8. CS^b^	.376**	.477**	−.024	.507**	.933**	.616**	.767**	–	
9. A	−.211**	−.218**	−.135**	−.141**	−.295**	−.253**	−.221**	−.283**	–
Mean	4.09	4.53	2.76	4.76	4.59	4.70	4.62	4.44	36.25
SD	0.35	0.47	0.87	0.37	0.55	0.52	0.57	0.82	4.57

Note: * *p* < .05, ** *p* < .01; *CS*^*a*^ Coping Strategy, *CC* Cognitive Coping, *EC* Emotional Coping, *BC* Behavioral Coping, *SS*^*a*^ Social Support, *SS*^*b*^ Subjective Support, *FS* Family Support, *CS*^*b*^ Counselor Support, *A* Anxiety

Confirmed as abnormally distributed data, Spearman’s correlation analyses were conducted. The results in Table 2 revealed that anxiety was significantly and negatively associated with coping (*r* = −.211, *p* < 0.01), and social support (*r* = −.295, *p* < 0.01). More precisely, the correlation coefficient between anxiety and social support (*r* = −.295, *p* < 0.01) was distinctly higher than that between anxiety and behavioral coping (*r* = −.141, *p* < 0.01). Notably, emotional coping was not significantly correlated with social support (*r* = −.022, *p* > 0.01).

### Mediation analyses

After finding internal links among coping strategy, social support, and anxiety, the study examined the potential mediating role of social support between coping strategy and anxiety. To obtain a detailed insight into coping strategy, the effect of the mediator variables was explored between the subscales of coping strategy (i.e., cognitive coping, emotional coping, and behavioral coping) and anxiety by bootstrapping. Since sex and academic attainment may serve as covariates, independent sample t-tests were used to compare group differences in the social support (M variable) and anxiety (Y variable). T-tests analyses showed that there were no significant differences in social support, the subscales of social support, and anxiety among students with various academic attainment (*p* > 0.05). The differences between male and female appeared insignificant in social support and the subscales of social support (*p* > 0.05), but significant in anxiety (t = − 4.347, *p* = 0.000). However, G*Power analyses showed that the effect size index *d* (Cohen’s *d* = 0.183) was smaller than 0.20, which indicated that the significant difference of sex in anxiety addressed little effect size [30]. Therefore, sex and academic attainment were not treated as covariates in the following mediation effect models.

**Fig. 2:**
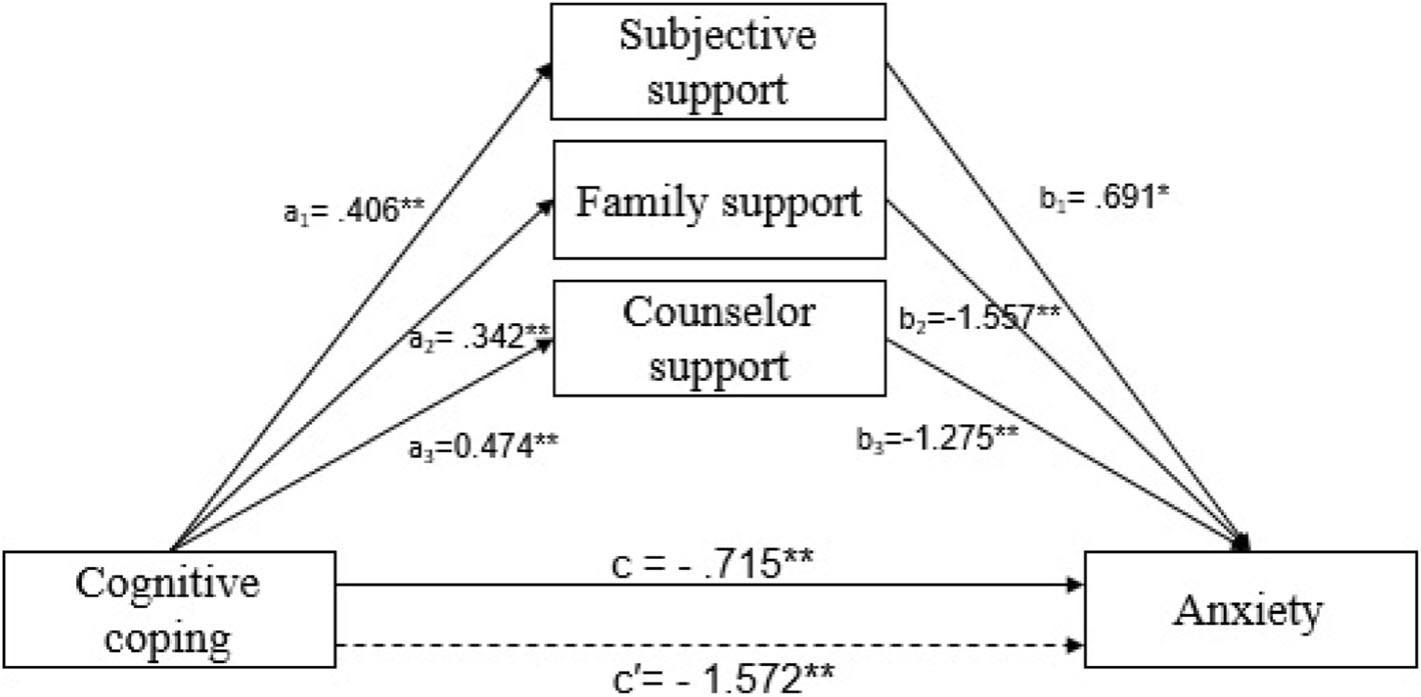
Note: All the coefficients are standardized; dashed lines indicate the total effect from cognitive coping to anxiety; * *p* < .05, ** *p* < .01

The mediating role of social support between cognitive coping and anxiety was evaluated first (see Fig. 2 and Table 3). In the direct effect model, before adding social support, cognitive coping negatively affected anxiety (c = − 0.715, *p* = 0.001). After the addition of social support, the indirect effect in the social support and anxiety pathway was negative and significant (c′ = − 1.572, *p* = 0.000), indicating that social support strengthened the negative effect of coping on anxiety. Furthermore, it is worth noting that subjective support positively and significantly affected anxiety (b_1_ = 0.691, *p* = 0.034). The path through the mediation of subjective support (point estimated = 0.28, 95% CI 0.011–0.62), family support (point estimated = − 0.533, 95% CI -0.799 – − 0.217), and counselor support (point estimated = − 0.604, 95% CI -0.923– − 0.419) were statistically significant, which supported H1.

**Table 3:** The mediation analysis of social support in the relationship between cognitive coping, behavioral coping and anxiety

**Model pathways**			***β***	***SE***	***t***	***P***	**LLCI**	**ULCI**
Direct effect X1 → Y		c	−.715	.211	−3.383	.001	−1.129	−.301
X1 → M1		a_1_	.406	.028	14.271	.000	0.35	0.462
X1 → M2		a_2_	.342	.021	15.948	.000	0.3	0.384
X1 → M3		a_3_	.474	.034	13.957	.000	0.408	0.541
M1 → Y		b_1_	.691	.326	2.117	.034	0.051	1.33
M2 → Y		b_2_	−1.557	.446	−3.492	000	−2.431	−0.683
M3 → Y		b_3_	−1.275	.142	−8.951	.000	−1.554	−0.996
Total indirect effect X1 → Y		c′	−1.572	.208	−7.564	.000	−1.98	−1.165
			**Effect**	**Boot** ***SE***	**Boot** **LLCI**	**Boot** **ULCI**	**Z**	***p***
Indirect effect	X1 → M1 → Y	a_1_ × b_1_	0.28	.163	0.011	0.62	1.724	.085
	X1 → M2 → Y	a_2_ × b_2_	−0.533	.178	−0.799	−0.217	−2.991	.003
	X1 → M3 → Y	a_3_ × b_3_	−0.604	.12	−0.923	−0.419	−5.047	.000
**Model pathways**			***β***	***SE***	***t***	***P***	LLC**I**	**ULCI**
Direct effect X2 → Y		c	.71	.283	2.507	.012	0.155	1.264
X2 → M1		a_1_	0.607	.036	16.915	.000	0.537	0.678
X2 → M2		a_2_	.634	.027	23.394	.000	0.581	0.687
X2 → M3		a_3_	.645	.043	15.058	.000	0.561	0.73
M1 → Y		b_1_	.691	.326	2.117	.034	0.051	1.33
M2 → Y		b_2_	−1.557	.446	−3.492	000	−2.431	−0 .683
M3 → Y		b_3_	−1.275	.142	−8.951	.000	−1.554	- 0.996
Total indirect effect X2 → Y		c′	−.681	.262	−2.595	.010	−1.195	- 0.167
			**Effect**	**Boot** ***SE***	**Boot** **LLCI**	**Boot** **ULCI**	**Z**	***p***
Indirect effect	X2 → M1 → Y	a_1_ × b_1_	0.42	.23	0.016	0. 944	1.827	.068
	X2 → M2 → Y	a_2_ × b_2_	−0.987	. 328	−1.557	− 0.383	−3.013	.003
	X2 → M3 → Y	a_3_ × b_3_	−0.823	. 117	−1.064	−0.608	−7.005	.000

Note: *N* = 2640. Number of bootstrap samples for bias-corrected bootstrap confidence intervals: 5000

Level of confidence for all confidence intervals: 95%. The top half is the results of the cognitive coping model, and the bottom half is the results of the behavioral coping model. *LLCI* lower level of the 95% confidence interval; *ULCI* upper level of the 95% confidence interval. *X1* cognitive coping, *X2* behavioral coping, *M1* subjective support, *M2* family support, *M3* counselor support, *Y* anxiety

**Fig. 3:**
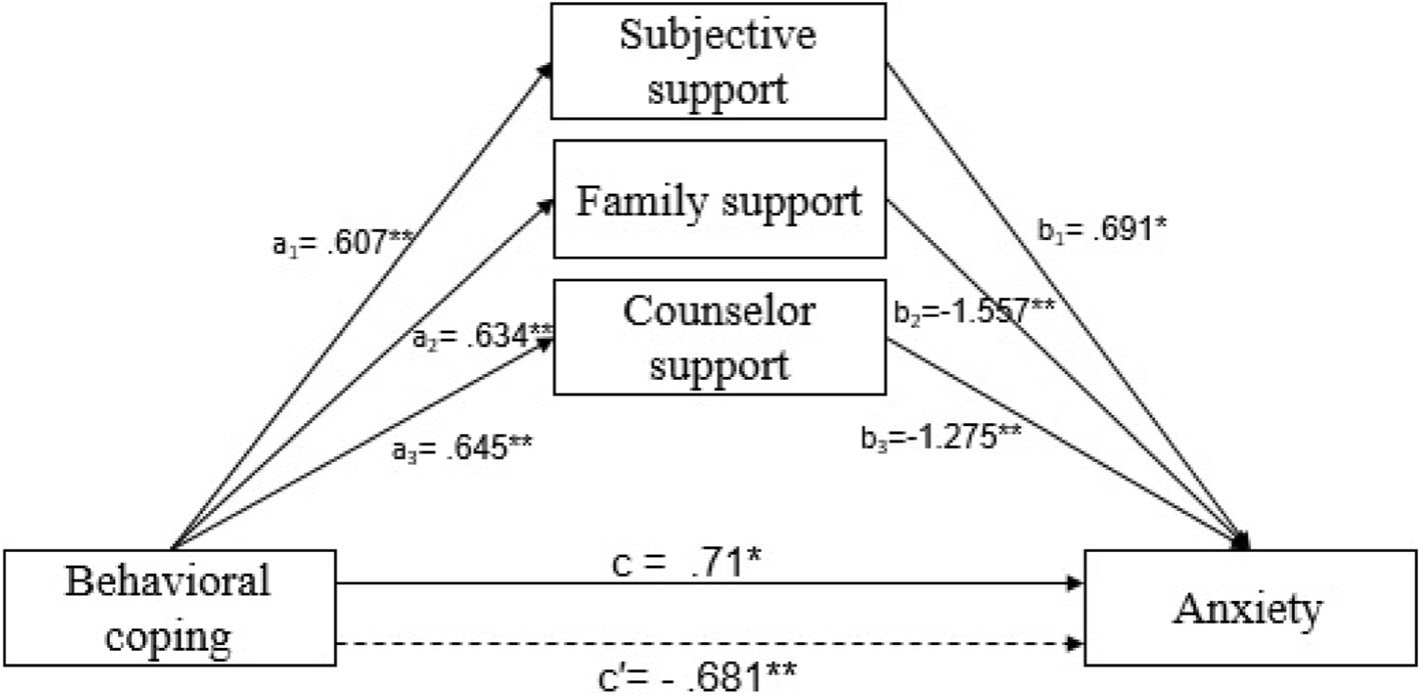
Note: All the coefficients are standardized; dashed lines indicate the total effect from behavioral coping to anxiety; * *p* < .05, ** *p* < .01

The mediating role of social support between behavioral coping and anxiety was then tested. As shown in Fig. 3 and Table 3, the total indirect effect (c′ = − 0.681, SE = 0.262, 95% CI -1.195 – − 0.167) of behavioral coping on anxiety was significant. The path through mediation of subjective support (point estimate =0.42; 95% CI 0.016–0.944), family support (point estimate = − 0.987; 95% CI -1.557 – − 0.383), and counselor support (point estimate = − 0.823; 95% CI -1.064 – − 0.608) were all statistically significant. Herein, the mediating effects of social support between behavioral coping and anxiety were significant at all social levels, which supported H3. The test also revealed that the positive effect of behavioral coping on anxiety could be reversed by the addition of social support, and then could negatively predict anxiety.

However, the mediation analysis of social support between emotional coping and anxiety indicates that the path through the mediation of subjective support (point estimate = 0.001; 95% CI - 0.023 – 0.017), family support (point estimate = − 0.001; 95% CI -0.035 – 0.032), and counselor support (point estimate = − 0.035; 95% CI -0.095 – 0.002) were all statistically insignificant, which did not support H2.

## Discussion

This study was conducted on a sample of Chinese college students to investigate the relationship between coping, social support, and anxiety during the COVID-19 pandemic. Two research questions and three hypotheses were proposed. The levels of Chinese college students’ coping strategies, social support, and anxiety were described, and a model of the relationship between coping strategies, social support, and anxiety was developed. Accordingly, some meaningful findings and implications were also drawn as follows.

First, this study found that the levels of cognitive coping, behavioral coping, and social support for Chinese college students were high while those of emotional coping were considerably lower. The finding of high cognitive coping is consistent with the finding that more than 90% of the Chinese public kept updating pandemic information [5]. However, this is not the case in 2009 H1N1 influenza survey, in which community awareness and perception of risk were not high [31]. What matters most is that college students are willing to follow the arrangement of the university and government and behave properly to prevent infection. The attached survey of this study showed that 97.05% of the college students supported the Chinese government’s strict lockdown measures, and the supporting rate of the closure of schools was 97.88%. The timely and transparent dissemination of scientific preventive information issued by the government and responsible university administrators, and efficient mechanism of countermeasures throughout the country have played dominant roles in guiding the students [25]. The high levels of social support are not consistent with the U.S. young-adult-sampled study demonstrating low perceived family support during the initial weeks of the pandemic [32]. It is possible that family cohesion, social networking, and ethnic identity serve as protective factors for mental status [33]. Behaviorally, the public is well-informed of the significance of the epidemic and strictly abides by hygiene guidance toward handwashing, mask wearing and social distancing [34]. Moreover, Chinese students’ innate and strong sense of solidarity and patriotism, and their unified trust in government are more likely to be exhibited when they confront crucial emergencies. The low emotional coping of Chinese students demonstrated their worries about the infection. This outcome is in line with the previous study indicating that 56% of respondents in the U.S. poll showed deep concerns about the dissemination of the coronavirus and a quarter of participants felt more nervous than they did during the 2014 Ebola pandemic [4]. According to a study on Weibo users, positive emotions declined after the outbreak of the pandemic [35]. Negative emotional reactions are universal under alarming circumstances.

Furthermore, the mean anxiety scores of the students were found to be higher than the Chinese norm of the SAS in the usual time, but much lower than those self-isolated in central China during the epidemic [7]. Similarly, according to a Chinese online survey from January 31st to February 2nd, 2020, moderate to severe anxiety symptoms appeared in 28.8% of the population [5]. Likewise, previous studies during SARS indicated that severe health emergencies could initiate anxiety [36]. Chinese comparatively low levels of anxiety may result from ethnic identities found in the recent pandemic report, indicating that Asian American young adults tended to experience low levels of anxiety compared to White counterparts [32].

Second, Spearman’s correlation analyses found that anxiety was significantly and negatively related to coping and social support and that social support possessed the greatest links with anxiety among these associations. This verified the dominating role of social support in buffering anxiety, which left room for further mediation analysis. There was a mediating effect of social support on the relationship between cognitive coping, behavioral coping, and anxiety, while social support did not mediate emotional coping and anxiety. Moreover, the path through family support and counselor support had stronger mediating power than subjective support. The influence of cognitive coping and behavioral coping on anxiety was mediated by social support, supporting H1 and H3. In the first path, cognitive and behavioral coping positively affected social support. Individuals with a sufficient input of information may be fully aware of the current epidemic situation, delivering an encouraging emotion to their family or themselves [37]. The constructive effect of behavioral coping on social support is probably because a majority of individuals cared for the potential risk for their family being infected and would remind their families to adopt hygiene precautions, which consequently strengthen the links among families [5]. In the second path, social support affected anxiety, with the positive effect of subjective support and the negative effect of family and counselor support. The finding of the prevailing role of family support over counselor support is similar to the pandemic study of U.S. young adults, stating that social support from family other than support from partners or friends could diminish the severity of mental illness [32]. Family support demonstrated a promising function to relegate anxiety in that family members were more likely to share mutual empathy and then social support took into effect emotionally [38]. Compared to individuals, family caregivers played a unique role in better articulating and supporting the emotional, social, and health needs of family members [39]. Further, counseling support could also bolster up the links. The effectiveness of counseling social support in prohibiting anxiety during the social-distancing period is addressed by the previous finding that videoconference, an alternative to in-person counseling, took effect in alleviating anxiety [40]. However, the finding of a positive effect of subjective support on anxiety contradicted the previous finding that patients with a high level of subjective social support tended to perceive fewer life stressors [41]. Subjective support of college students, namely, individual resilience during the pandemic, was associated with high levels of anxiety, considering that they may be young adults and have less capacity to struggle with intense fear and anxiety. The finding that the mediating impact of social support did not exist in emotional coping and anxiety, which contradicted H2, could be probably inferred that emotional coping affected anxiety directly. This is consistent with a prior study on SARS, demonstrating that the symptoms of posttraumatic stress, anxiety, and depression appeared simultaneously in surviving patients [42].

Overall, the finding of the positive mediating effect of social support is of great value because it provides empirical evidence to emphasize the implementation of social support. Therefore, it is urgent to take measures to facilitate social support during the COVID-19 pandemic. For instance, regarding the information about infectious diseases, individuals should avail themselves of the suggestions of professional medical staff [43]. Second, students should communicate more frequently with families, relatives, and friends by phone or via the internet [44]. Under the circumstance of isolation, the use of online networks can augment social support, sustaining the mutual power of online social groups [45]. More importantly, it is indispensable to acquire the engagement of social workers and psychotherapists when individuals are confronted with mental burdens [25]. Third, since routine face-to-face psychological counseling is apparently unavailable during the epidemic period, remote counseling, such as structured letter therapy, e-mail, or other telemediated alternative means of communication, can be a feasible psychological intervention solution to diagnose and treat [46]. In addition, most Chinese university counseling centers could provide hotlines for psychological interventions and online platforms for psychoeducation, such as training programs and curricula, which can be easily accessible for students via WeChat and Tencent [44]. Under the university and community setting, free counseling training clinics and departments of student affairs should be advocated to provide adequate preparation for easing mental distress [47]. To highlight the significance of counselor support, university psychological professionals should harness digital technologies to conduct online counseling or life education during the pandemic, which may be perceived as innovative teaching approaches [25].

### Limitations

However, there are some limitations to the current study. First, the samples were restricted to one university, which may be less generalizable to the entire population. Second, the cross-sectional design of this study could not reflect the long-term development of the samples, which needed supplementary findings from longitudinal research. In addition, all the questionnaires were self-reported, and the veracity of the result relied on the self-perception of the subjective and non-subjective methods should be applied. Last but not least, anxiety as a dependent variable involved in the survey could not reflect an intact picture of mental health, and it is necessary to further explore other factors indicating traumatization, such as depression, fear, despair, and irritability.

## Conclusion

The findings in this empirical study are supposed to present a clue in psychological interventions and behavioral guidance on the current global crisis. First, this study detected high levels of cognitive coping, behavioral coping, and social support, explored low levels of anxiety and emotional coping for Chinese college students at the peak of the COVID-19 pandemic from January to February 2020. Second, anxiety was negatively associated with coping and social support. Third, social support played as a mediator in the relationships between cognitive coping, behavioral coping, and anxiety, with family support and counselor support exerting a stronger negative power against anxiety than subjective support.

In view of the implications proposed, there appear to be alternate strategies to ease anxiety. These findings may provide evidence for promoting social support, especially family support and counselor support, as facilitating sources to decrease anxiety in coping with the COVID-19 pandemic cognitively and behaviorally. Policymakers and school administrators should encourage meaningful communication between family members and activate effective counseling services to maintain positive mental health.

## Supplementary Information

**Additional file 1: Table S1.** Items of Coping Strategy Questionnaire (CSQ).

## Data Availability

The data that support the findings of this study are not currently publicly available due to institutional regulations protecting service member survey respondents but are available from the corresponding author on reasonable request (may require data use agreements to be developed).

## Abbreviation

COVID-19: Corona Virus Disease 2019
