# Supplementary material for: Does social support matter? The mediating links with coping strategy and anxiety among Chinese college students in a cross-sectional study of COVID-19 pandemic
Source: BMC Public Health. 2021 Jul 2;21:1298. doi: 10.1186/s12889-021-11332-4 (PMC8253469; doi:10.1186/s12889-021-11332-4)
Supplement: Supplementary file 1 — Additional file 1: Table S1. Items of Coping Strategy Questionnaire (CSQ). [file 12889_2021_11332_MOESM1_ESM.doc]

Additional file 1：

Table S1. Items of Coping Strategy Questionnaire (CSQ)

| Items |
| --- |
| 1. I understand the preventive information regarding COVID-19. |
| 1. I have paid close attention to the news about the COVID-19 epidemic. |
| 1. I believe that the COVID-19 epidemic can be under effective control. |
| 1. I am worried about being infected with COVID-19. |
| 1. I am afraid that everyone could be infected with COVID-19. |
| 1. I feel unsafe even if I wear mask outside. |
| 1. I wear mask when I go outside. |
| 1. I have paid more attention to the personal health than before. |
| 1. I have cancelled the schedule of outing and social gathering. |
| 1. I have tried to avoid going to the hospital to keep away from infection. |
